# Reconstructing a comprehensive transcriptome assembly of a white-pupal translocated strain of the pest fruit fly *Bactrocera cucurbitae*

**DOI:** 10.1186/s13742-015-0053-x

**Published:** 2015-03-31

**Authors:** Sheina B Sim, Bernarda Calla, Brian Hall, Theodore DeRego, Scott M Geib

**Affiliations:** 1 Tropical Crop and Commodity Protection Research Unit, Daniel K. Inouye US Pacific Basin Agricultural Research Center, USDA Agricultural Research Services, Hilo, HI USA; 2 Department of Plant and Environmental Protection Sciences, University of Hawaii, Manoa, Honolulu, HI USA

**Keywords:** *Bactrocera cucurbitae*, Translocation, RNA-Seq, White-pupae, Tephritidae, Melon fly, SIT, Sterile insect technique

## Abstract

**Background:**

*Bactrocera cucurbitae* is a serious global agricultural pest. Basic genomic information is lacking for this species, and this would be useful to inform methods of control, damage mitigation, and eradication efforts. Here, we have sequenced, assembled, and annotated a comprehensive transcriptome for a mass-rearing sexing strain of this species. This forms a foundational genomic and transcriptomic resource that can be used to better understand the physiology and biochemistry of this insect as well as being a useful tool for population genetics.

**Findings:**

A transcriptome assembly was constructed containing 17,654 transcript isoforms derived from 10,425 unigenes. This transcriptome size is similar to reports from other Tephritid species and probably includes about 70-80% of the protein-coding genes in the genome. The dataset is publicly available in NCBI and GigaDB as a resource for researchers.

**Conclusions:**

Foundational knowledge on the protein-coding genes in *B. cucurbitae* will lead to improved resources for this species. Through comparison with a model system such as *Drosophila* as well as a growing number of related Tephritid transcriptomes, improved strategies can be developed to control this pest.

## Data description

### Background

*Bactrocera cucurbitae* (Diptera: Tephritidae) is an important agricultural pest attacking many fruits and vegetables in tropical and subtropical regions. To maximize efforts to control, mitigate the damage, and maintain eradication of this invasive species in the mainland United States, we must accumulate foundational information that describes the many aspects of the biology of this species. The data we present is a comprehensive transcriptome of the T1 translocated sexing strain of *B. cucurbitae* and represents genes expressed across all major life stages [1]. The white pupae trait of this line is sex-linked and makes this strain conducive to large-scale rearing and male-only mass release of sterilized flies for the sterile insect technique (SIT).

### Samples

Samples were derived from the T1 *white pupae* translocated line of *B. cucurbitae* maintained at the USDA-ARS Daniel K. Inouye Pacific Basin Agricultural Research Center Insectary in Hilo, Hawaii, USA [1]. To generate samples that are representative of a broad range of life stages and ages, daily samples were collected from eggs (0–2 days old), larvae (~0-10 day post-hatch), pupae (0–10 days post-pupation) and adults (both unmated and mated males and females) as previously described [2]. Total RNA was extracted from samples across each stage, and then representative, stage-specific samples were generated through pooling to generate four RNA samples for sequencing which are described as NCBI BioSamples SAMN03010448- SAMN03010451 associated with BioProject PRJNA259566. For each sample, RNA was extracted using the Zymo Quick-RNA miniprep extraction kit (Zymo Research, Irvine, CA) following recommended procedures for whole-tissue extraction. The resulting RNA was quantified with the Qubit Broad Range RNA assay on a Qubit 2.0 fluorimeter (Life Technologies, Carlsbad, CA), and size and quality determined with an RNA 6000 nano chip on an Agilent 2100 Bioanalyzer (Agilent Technologies, Santa Clara, CA).

### Sequencing

Total RNA was sent to the Beijing Genomics Institute (BGI Americas, at University of California, Davis, USA) and eukaryotic mRNA libraries were prepared using TruSeq technology (TruSeq RNA Kit v2). The resulting four libraries (egg, larvae, pupae, and adult) were barcoded and sequenced together on a single lane of Illumina HiSeq 2000, generating approximately 39.31 Gb of raw data from approximately 196 million 2 × 100 bp paired reads. These raw reads were filtered by quality and for adaptor contamination using an in-house pipeline at BGI, targeting reads containing adaptor, reads with more than 5% ambiguous bases, or reads with greater than 50% of bases with Phred quality score below 10. This reduced the reads to approximately 37.78 Gb after filtering, removing approximately 4% of the data. This filtered data was used as the input to the *de novo* assembly and also deposited at GenBank under the SRA accessions SRS691531- SRS691534.

### Transcriptome assembly

A single representative *de novo* assembly was generated from a concatenation of the four libraries using the Trinity pipeline (r2014_07-17) [3,4]. In brief, read abundance was normalized *in silico* to 50X coverage, and then assembled using default Trinity parameters, with the exceptions of the addition of the ‘--jaccard_clip’ flag to reduce the generation of transcript fusions from non-strand specific data. After assembly, transcript and unigene level expression values were calculated using RSEM [5] and open reading frames (ORFs) were predicted with Transdecoder [4]. In addition to Transdecoder-predicted ORFs, ORFs were included that had a Pfam-A domain match utilizing Hmmer3 to perform searches [6]. Next, the raw transcriptome was filtered to discard poorly supported transcripts and maintain transcripts with strong evidence for protein-coding regions and reasonable support for being expressed. Transvestigator was implemented [7], and parameters were set to retain only those transcripts that have a transcript per million (TPM) value greater than 0.5, that at an isoform level represent at least 5% of the read abundance based expression for the parent unigene, and that have a predicted ORF. A similar filtering strategy was used for two previously published Tephritid transcriptomes [2,8], and should allow similar quality transcriptomes and comparison between these species. In addition to filtering the transcriptome, Transvestigator also prepared the dataset for NCBI Transcriptome Shotgun Assembly (TSA) submission by ensuring the predicted ORF is in the positive strand, confirming presence of only a single ORF per transcript, and generating a properly formatted NCBI .tbl file for submission. Details on transcriptome assembly and annotation statistics are listed in Table 1.

**Table 1:** **Transcriptome assembly and annotation statistics compared with other Tephritid transcriptomes and the** ***Drosophila melanogaster*** **genome**

Species	*B. cucurbitae*	*B. dorsalis* ^*a*^	*C. capitata* ^*b*^	*D. melanogaster* ^*c*^
Number of read pairs used in assembly (SRA accession number)				
Egg (SRA: SRS691534)	43741314	12462204	-	-
Larvae (SRA: SRS691533)	51568835	11753084	-	-
Pupae (SRA: SRS691532)	47093178	13291147	93256673	-
Adult (SRA: SRS691531)	46515243	47250123	96929532	-
Total	188918570	84756558	190186205	-
Normalized reads (*in silico* normalization)	12792085	7796491	17217414	-
**Unfiltered assembly**				
Number of unigenes (or *Drosophila* genes)	50220	47216	118793	-
N50 longest transcript/unigene	2191	1882	1187	-
Sum longest transcript/unigene (Mb)	49.63	40.20	81.56	-
Number of transcripts	76688	80345	190958	-
N50 transcript length (bp)	2626	2802	2686	-
Sum transcript length (Mb)	100.20	109.48	236.18	-
Transcripts per unigene	1.53	1.70	1.61	-
GC %	38.10	39.11	36.21	-
**Filtered** ***de novo*** **assembly or current** ***Drosophila*** **release**				
Number of unigenes	10425	10784	10741	15504
N50 unigene length (longest transcript/unigene)	3464	3043	3383	2979
Sum longest transcript/unigene (Mb)	28.12	24.46	28.34	30.53
Number of transcripts	17654	23539	21761	25205
N50 transcript length (bp)	3477	3460	3913	3633
Sum transcript length (Mb)	48.28	62.06	66.65	68.47
Isoforms per unigene	1.69	2.18	2.03	1.63
GC %	40.17	40.32	39.41	49.70
N50 protein length (amino acids)	323	301	310	370
Number of proteins with complete ORF (%)	12936 (73.2)	13017 (55.3)	15740 (72.3)	-
**Annotation statistics**				
Number of proteins with PFAM domains identified	13029	16612	13646	-
Number of proteins with Gene Ontology Terms	10640	-	13648	-
Number of proteins with gene names	15956	17093	15841	-
Number of proteins with significant hit to *Drosophila* proteins^d^	16070	20713	19245	-

^a^Data from Geib et al., 2014 [2]; ^b^Data from Calla et al., 2014 [8]; ^c^Data from Flybase r6.03 [11]; ^d^BLASTP hit with e-value cutoff 1e-5.

### Annotation

Annotation was performed at the peptide level, and annotations were used to generate a transcript name and product in addition to functional annotations. All predicted proteins were subjected to analysis through InterProScan5, searching all available databases including Gene Ontology and InterPro term lookup [9]. In addition, proteins were subjected to a BLASTP search against the UniProt SwissProt database (downloaded 10 November 2013). Transcripts were annotated with UniProt and InterProScan results using Annie [10], a program that extracts qualified gene names and products by cross-referencing SwissProt BLAST hits and performs database cross-referencing from InterProScan5 results. The resulting annotation file was provided to Transvestigator, described above, to include functional annotations in the resulting.gff3 and .tbl files. The filtered and annotated transcriptome was deposited at GenBank as a TSA under the accession GBXI00000000 associated with BioProject PRJNA259566. Annotation statistics are listed in Table 1.

### Comparison of *B. cucurbitae* transcriptome with other published datasets

**Figure 1:**
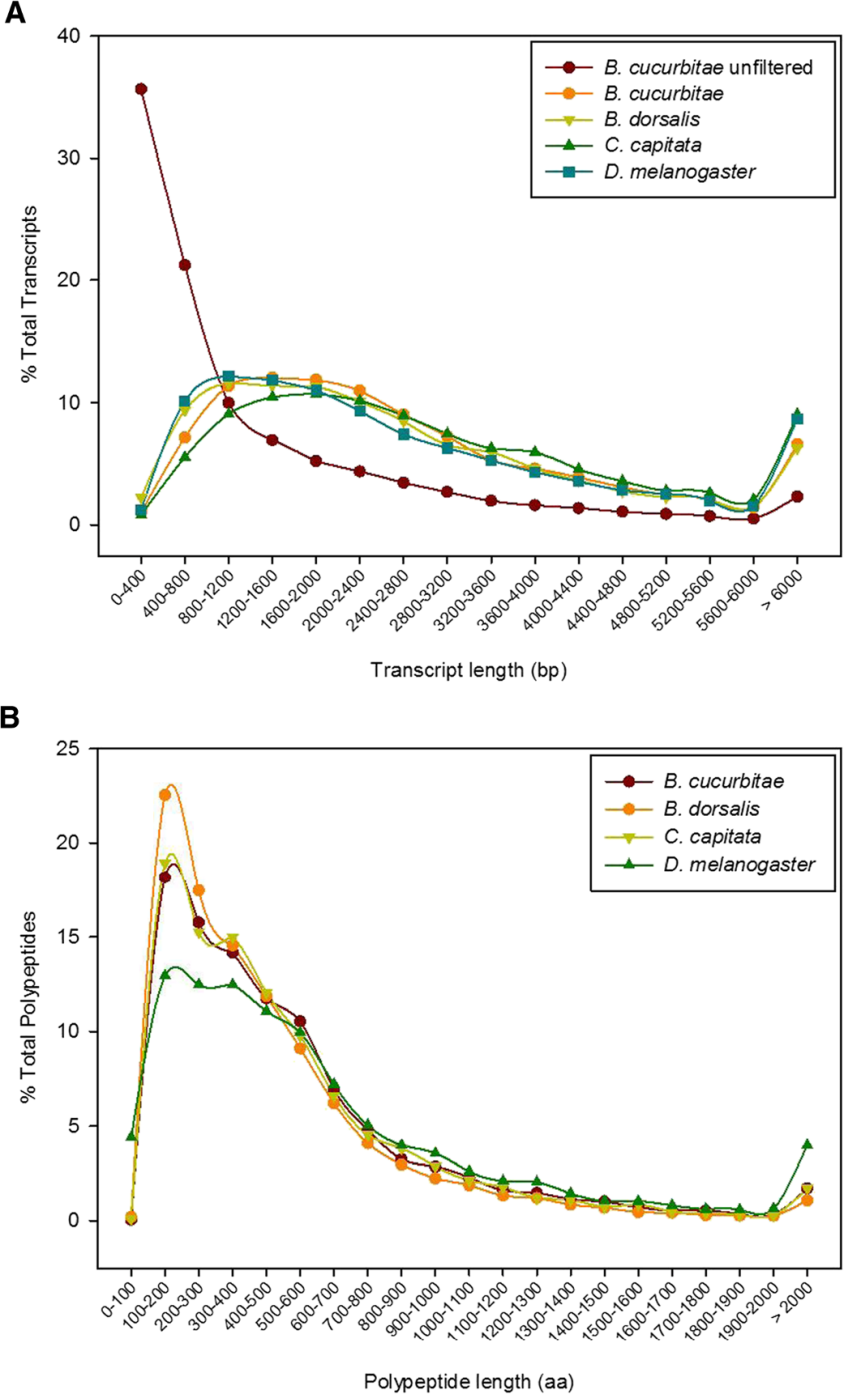
**Comparison of*B. cucurbitae*transcriptome to related fly species.** Distribution of **(A)** transcript length and **(B)** predicted polypeptide length of the *B. cucurbitae* transcriptome compared with published *Bactrocera dorsalis* and *Ceratitis capitata de novo* transcriptome assemblies and the current *Drosophila melanogaster* transcript/protein set (Flybase r6.03).

Two previously published *de novo* Tephritid transcriptomes (*Bactrocera dorsalis* [2] and *Ceratitis capitata* [8], as well as the current *Drosophila melanogaster* genome transcript and peptide datasets (Flybase.org r6.03) [11] were used to compare the relative quality and completeness of the *B. cucurbitae* transcriptome. Figure 1 displays a histogram distribution of transcript length and predicted peptide length for all four species, in addition to the raw unfiltered *B. cucurbitae* transcriptome dataset. This demonstrates that the relative distribution of transcript length is consistent with what is seen in other Tephritid species and also, the Tephritid distribution is consistent with what is seen in *Drosophila*. In addition, the majority of the filtered transcripts fall outside of the expected distribution, supporting their removal from the assembly. Looking at summary assembly statistics (Table 1), unigene and transcript abundance is similar to other Tephritid transcriptomes, and the proportion of transcripts that could be functionally annotated is similar across species. Based on these comparisons, the *B. cucurbitae* transcriptome presented here is of high quality, and should serve as a foundational resource to promote molecular and biochemical research on this important pest species.

## Availability of supporting data

The filtered and annotated transcriptome has been deposited at GenBank as a transcriptome shotgun assembly (TSA) under the accession GBXI00000000 associated with BioProject PRJNA259566. Supporting data and analysis results also available from the *GigaScience* GigaDB database [12].
